# Sexithiophenes as efficient luminescence quenchers of quantum dots

**DOI:** 10.3762/bjoc.7.202

**Published:** 2011-12-22

**Authors:** Christopher R Mason, Yang Li, Paul O’Brien, Neil J Findlay, Peter J Skabara

**Affiliations:** 1 Department of Chemistry, University of Manchester, Oxford Road, Manchester, M13 9PL, UK; 2 WestCHEM, Department of Pure and Applied Chemistry, University of Strathclyde, Glasgow, G1 1XL, UK

**Keywords:** electrochemistry, luminescence quenching, quantum dots, sexithiophenes, synthesis

## Abstract

Sexithiophenes **1a** and **1b**, in which a 4-(dimethylamino)phenyl unit is incorporated as an end-capping group, were synthesised and characterised by cyclic voltammetry, absorption spectroscopy and UV–vis spectroelectrochemistry. Additionally, their ability to function as effective luminescence quenchers for quantum dot emission was studied by photoluminescence spectroscopy and compared with the performance of alkyl end-capped sexithiophenes **2a** and **2b**.

## Introduction

**Figure 1:**
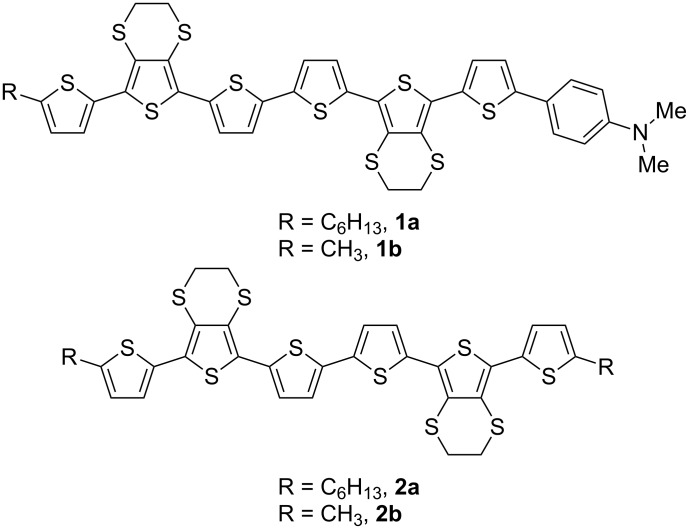
Dimethylaminophenylene end-capped sexithiophenes **1a** and **1b**, and dialkyl end-capped sexithiophenes **2a** and **2b**.

Structurally well-defined oligothiophenes as functional organic materials have attracted significant interest due to the advantages they offer over polythiophenes, namely (i) monodispersity, (ii) a precise structure with no isomeric impurities, (iii) high chemical stability, (iv) good solubility, and (v) direct processability from solution or by vacuum deposition [1]. This has led to the application of oligothiophenes in numerous organic devices including solar cells [2–4], light-emitting diodes (LEDs) [5–6], field-effect transistors [7–9] and electrochromics [10]. Furthermore, end-capped oligothiophenes are particularly attractive materials for study due to their enhanced stability, and this can lead to improved performance in devices through enhanced intermolecular ordering [11]. Tailoring of the properties of oligothiophenes can be achieved by the appropriate choice of the end-capping functionality, for example, the incorporation of perfluoroalkyl groups for n-type semiconductors [12–13]. Previously, we reported the synthesis and properties of alkyl end-capped oligothiophenes **2** (Figure 1), which incorporate the ethylene dithiothiophene (EDTT) unit, including their performance as the electron donor material in a bilayer photovoltaic device [14]. In this article, the role of dimethylamino end-capping groups in coordinating to nanocrystalline particles is reported.

Recently, there has been significant interest in small, nanocrystalline particles, or quantum dots, from both a fundamental point of view, and also with regards to their potential role in a range of device architectures [15–16]. Additionally, the formation of blends of conjugated oligomers and quantum dots can lead to attractive properties, taking advantage of facile charge transfer due to the high electron affinity of the quantum dot nanoparticles [17–18], as well as overcoming processing difficulties associated with devices containing quantum dots alone [15]. The complementary properties of conjugated oligomers/polymers and nanoparticles, and the possible photoinduced electron-transfer processes between them, have led to these materials being combined successfully in LEDs and photovoltaic cells [19–23]. Previously, we reported the synthesis and characterisation of CdS quantum dots in polystyrene beads, in which beads ranging in size from 100 nm to 500 μm were prepared and confocal microscopy showed an even distribution of CdS throughout the polymer, with retention of the photoluminescence behaviour of the quantum dot [24]. Herein, we present a comparative study on the photophysical properties of sexithiophenes **1** or **2** in varying concentrations, in the presence of a fixed concentration of CdSe(ZnS) core/shell quantum dots. Our choice of compounds represents molecules with (**1a**,**b**) and without (**2a**,**b**) a Lewis base functionality for quantum-dot surface coordination, with a view to determining whether structural complexity is required to achieve nanoparticle–sexithiophene electronic interactions.

## Results and Discussion

### Synthesis

**Scheme 1:**
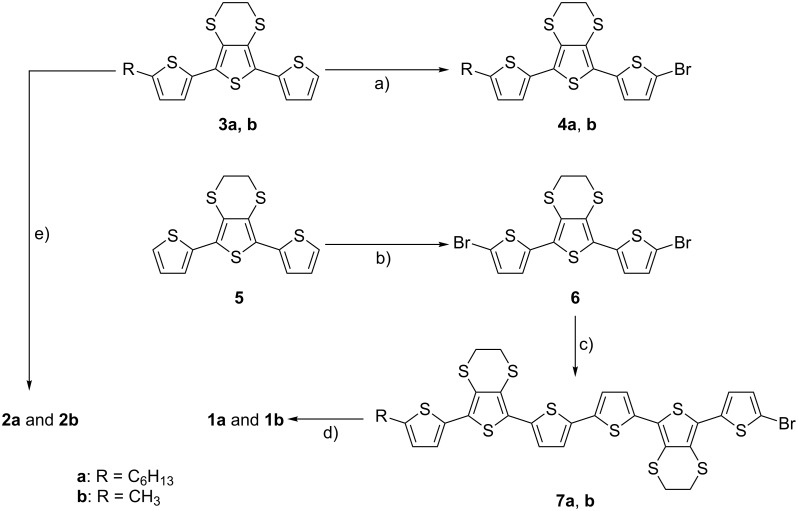
The synthesis of functionalised oligothiophenes **1a**,**b** and **2a**,**b**. Reagents and conditions: a) NBS, CH_3_CO_2_H, THF (1:1, v/v); **4a**, 97%, **4b**, 88%; b) 2.2 equiv NBS, CH_3_CO_2_H, THF (1:1, v/v); **6**, 91%; c) (i) *n-*BuLi, (ii) ZnCl_2_, (iii) **4a** or **4b**, Pd(PPh_3_)_4_, THF; **7a**, 40%, **7b**, 20%; d) 4-(dimethylamino)phenylboronic acid, Pd(PPh_3_)_4_, toluene, EtOH, NaHCO_3_, H_2_O; **1a**, 28%, **1b**, 44%; e) FeCl_3_, CHCl_3_; **2a**, 40%, **2b**, 50%.

The preparation of sexithiophenes **1a** and **1b** (Scheme 1) began in a similar fashion to our previously published methodology for the synthesis of compounds **2a** and **2b** [14]. After the formation of terthiophenes **3** [14], bromination with NBS under acidic conditions afforded the key intermediates **4a** and **4b** in high yields (97% and 88%, respectively). In parallel, dibrominated terthiophene **6** was prepared in an analogous fashion to **4a** and **4b** with 2.2 equiv of NBS. Subsequent Negishi coupling of compound **6** with organozinc intermediates of **4a** and **4b**, which were prepared by lithiation followed by reaction with zinc chloride, led to the isolation of **7a** and **7b** in modest yields (40% and 20%, respectively). Sexithiophenes **1a** and **1b** were subsequently isolated following Suzuki–Miyaura coupling with 4-(dimethylamino)phenylboronic acid (**1a**, 28%; **1b**, 44%). As a comparison, nonfunctionalised sexithiophenes **2a** and **2b** were also synthesised in order to study the role of the dimethylaminophenyl group on the oligothiophene properties. The synthesis of **2a** and **2b** was completed by following our published procedure [14], with oxidative coupling of **3a** or **3b** with the aid of FeCl_3_ affording sexithiophenes **2a** and **2b** in moderate yields (40% and 50%, respectively).

### Characterisation of physical properties

**Figure 2:**
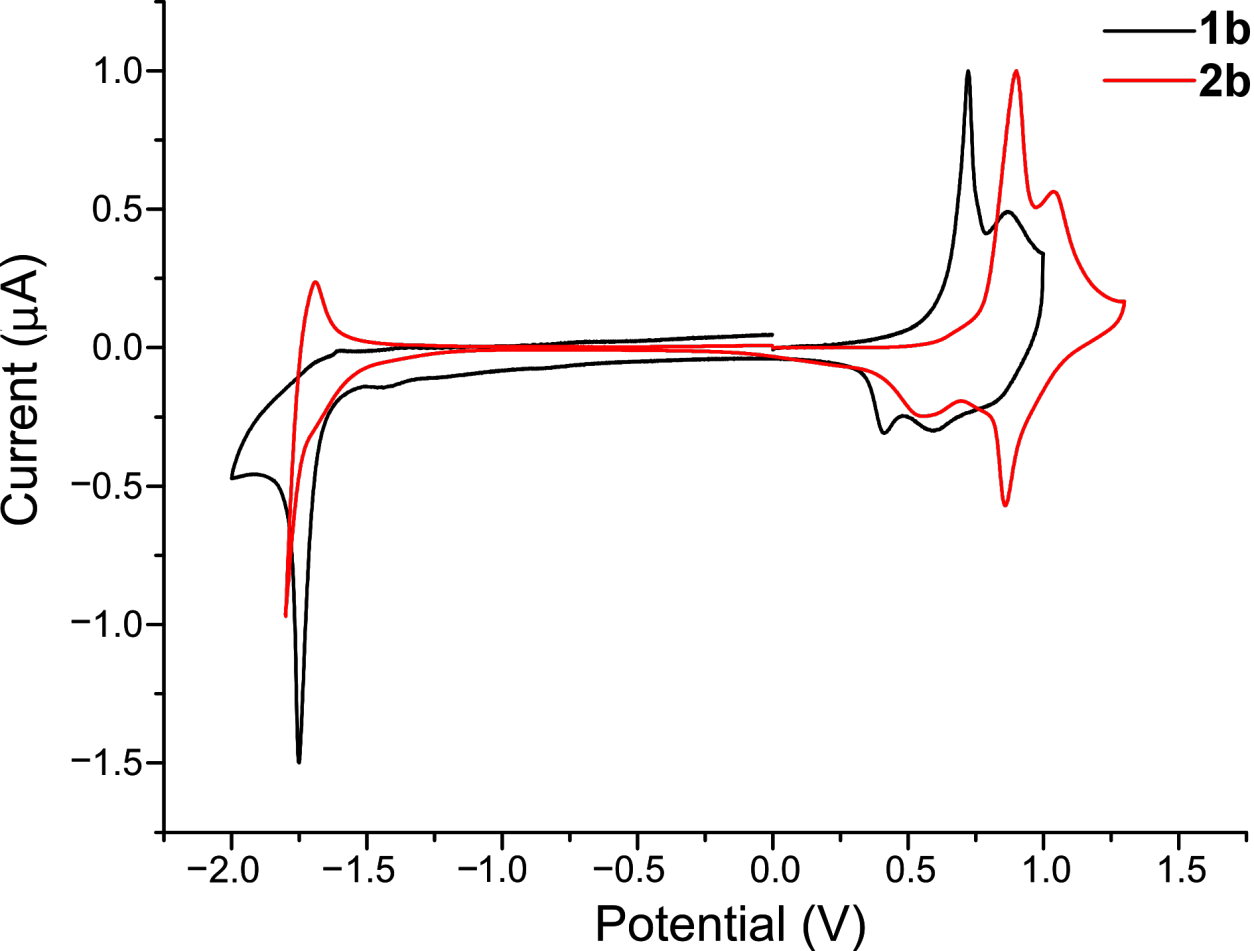
Solid-state voltammograms of **1b** and **2b**, as spin-coated films on ITO glass, versus Ag/AgCl reference electrode, platinum wire as the counter electrode, TBAPF_6_ as the supporting electrolyte in CH_3_CN (0.1 M), scan rate 100 mV s^−1^.

A film of methyl-capped dimethylaminosexithiophene **1b** on ITO glass was obtained by spin coating from a chloroform solution, and the redox properties were compared to those of nonfunctionalised analogue **2b** (Figure 2; Table 1).

**Table 1:** Redox and peak separation potentials of **1b** in the solid state compared to **2b**. The HOMO–LUMO gap was determined from the difference in the onsets for the reduction and first-oxidation processes.

Entry	*E*_1_^½^ (V)	Δ*E*_1a–c_ (mV)	*E*_2_^½^ (V)	Δ*E*_2a–c_ (mV)	HOMO–LUMO gap (eV)

**2b**	+0.73	340	+0.95	170	2.2
**1b**	+0.56	290	+0.74	270	2.1

In the positive scan, the cyclic voltammogram for **1b** shows two quasi-reversible redox waves consistent with the formation of a polaronic cation at the first step followed by the formation of the dication species at the second oxidation. Compared to **2b**, the first oxidation peak of **1b** is shifted to a less positive value, viz. from +0.90 V to +0.70 V. The corresponding reduction process is also shifted from +0.56 V to +0.41 V. The second oxidation potential for **1b** experiences a shift to lower values (1.04 V compared to 0.89 V), but the reversibility is diminished somewhat; Δ*E*_2a–c_ (mV) **2b**, 170 mV; **1b**, 270 mV. The decrease in oxidation potentials of **1b** compared to those of **2b** indicates a more effective stabilisation or accommodation of the positive polaron and bipolaron species in **1b**, due to the addition of the electron-donating dimethylaminophenyl substituent. In the negative scan, similar reduction potentials were observed at −1.75 and −1.80 V for **1b** and **2b**, respectively. The electrochemical band gap for **1b** in the solid state is approximately 2.1 eV, which is slightly lower than that of **2b** (2.2 eV) due to the electronic contribution of the conjugated phenylamine.

**Figure 3:**
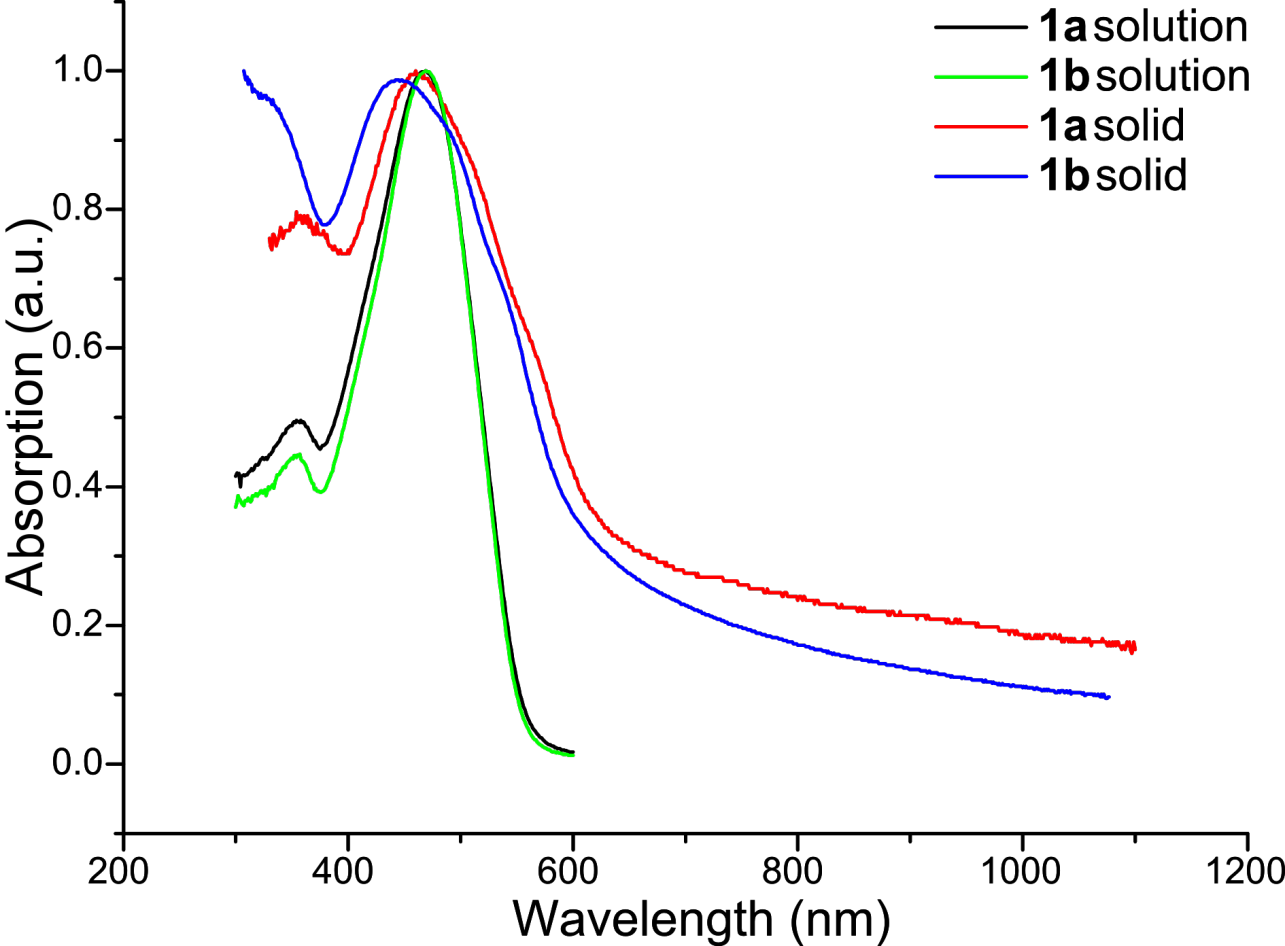
Absorption spectra in solution (dichloromethane) and solid state.

Absorption studies (in dichloromethane) on functionalised sexithiophenes **1a** and **1b** revealed a bathochromic shift in the absorption maximum compared to the corresponding dialkyl end-capped sexithiophenes **2a** and **2b** (shifts of 10 and 12 nm for compounds **1a** (λ_max_ = 468 nm) and **1b** (λ_max_ = 469 nm), respectively (Figure 3; Table 2) [14]). These bathochromic shifts are consistent with the increase in π-electron delocalisation due to the addition of the conjugated phenyl ring. This increase in conjugation is also evidenced by a slight reduction in the optically determined HOMO–LUMO gap of **1a** and **1b** (2.2 eV for both, Table 2). The solid-state absorption spectra of **1a** and **1b** are very similar, but the optical HOMO–LUMO gaps in the solid state are red-shifted compared to those in solution (2.0 eV).

**Table 2:** Comparison of the optical properties of **1a**, **1b**, **2a** and **2b** in solution and solid state. The HOMO–LUMO gap was determined from the onset of the longest-wavelength absorption band.

Entry	Absorption maxima (nm)	HOMO–LUMO gap (eV)

**1a**	468	2.2
**2a**	458	2.3
**1a** solid	469	2.0
**1b**	469	2.2
**2b**	457	2.3
**1b** solid	467	2.0

**Figure 4:**
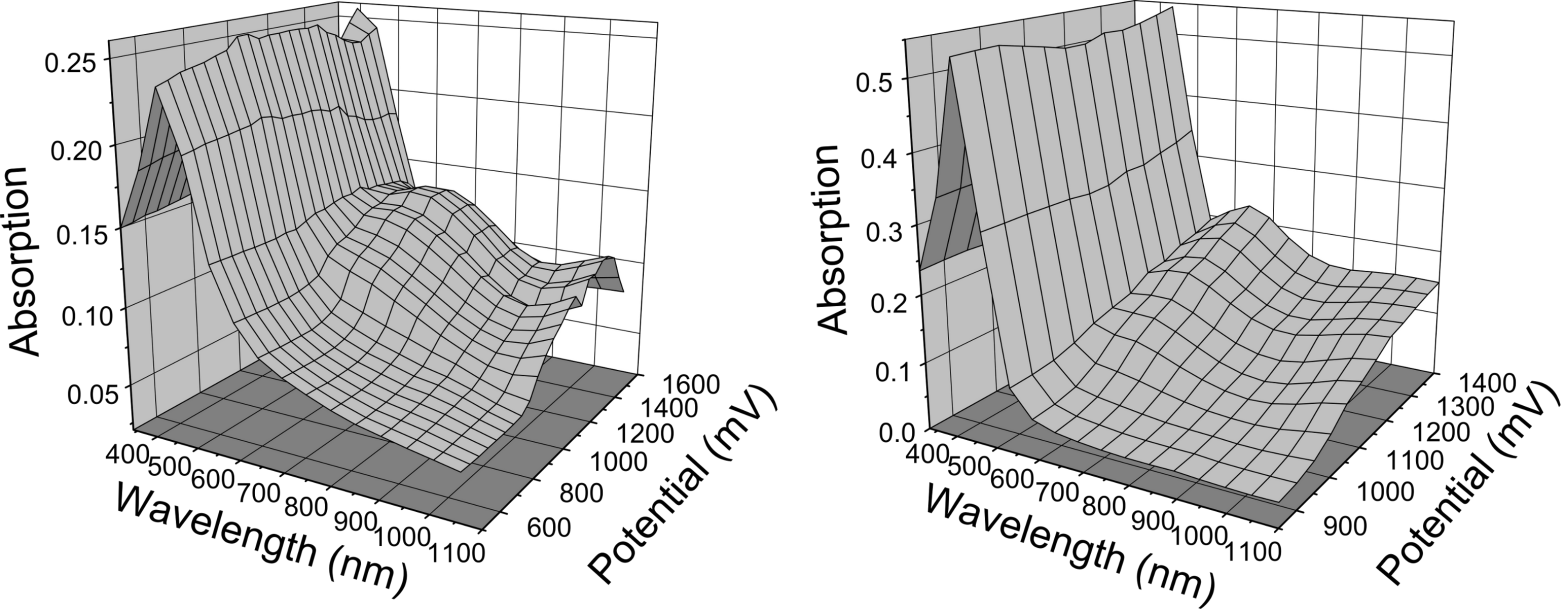
UV–visible spectroelectrochemical measurements of **1b** (left) and **2b** (right) drop-cast onto ITO glass.

UV–vis spectroelectrochemical measurements (SEC) were performed in acetonitrile on functionalised sexithiophene **1b** and nonfunctionalised sexithiophene **2b** as thin films drop-cast onto ITO glass (Figure 4). The methyl derivatives were used in this study since the hexyl analogues were found to dissolve partially, upon oxidation and reduction. In this context, it should be noted that the difference between the spectroelectrochemical behaviours of the methyl and hexyl sexithiophenes is expected to be negligible. During the experiments, no new peaks appeared in either sample until the second oxidation process. At this point, two new peaks emerge above +0.80 V at 668 and >1100 nm for **1b** and are assigned to the dication, as are both new peaks in **2b** (at 651 and >1100 nm). These continue to grow up to +1.50 V until a sharp decline in absorption is seen due to the degradation or dissolution of the films (Figure 4, compound **1b**). Intermediate polaron peaks were not seen for either **1b** or **2b**. The difference between the first and second oxidation potentials (0.19 V) for **1b** is slightly larger than that for **2b** (0.13 V) but the peaks may still be sufficiently close enough together to prevent the experimental detection of the intermediate polaron (**1b**^+·^). The increase in wavelength of the high-energy absorption band of **1b**^2+^ (668 nm) compared to that of **2b**^2+^ (631 nm) indicates a more delocalised charged species. This increase is a consequence of the resonance effect of the phenyldimethylamine group.

### Sexithiophene/quantum dot composites in solution

**Figure 5:**
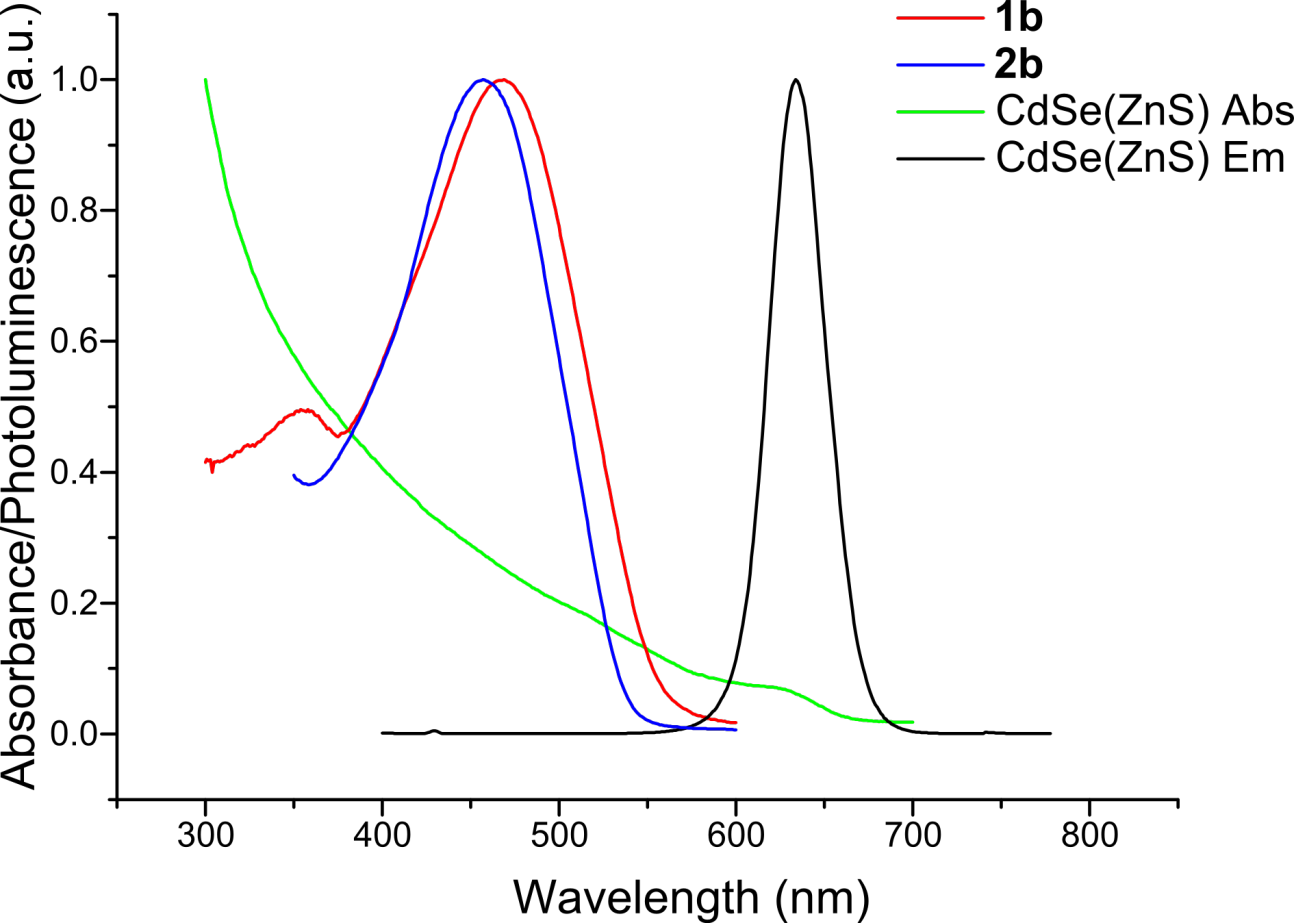
Absorption spectra for **1b** and **2b**, together with the absorption and emission profiles for the CdSe(ZnS) quantum dots, all in chloroform.

A comparative study on the photophysical properties of sexithiophenes **1a**,**b** and **2a**,**b**, in varying concentrations, with a fixed concentration of CdSe(ZnS) core/shell quantum dots was conducted to determine the effect of the Lewis base group in sexithiophenes **1a** and **1b**. The quantum dot CdSe core was prepared by the method of Cumberland et al. [25], and the ZnS shell was added using the dithiocarbamate precursor Zn(S_2_CNMeHex)_2_ [26]. The resulting dots incorporate a hexadecylamine (HDA) capping group and were prepared so as to ensure that their fluorescence did not overlap with the absorption profile of the sexithiophenes under study. Thus, the photoluminescence maximum for these quantum dots under excitation of light at 590 nm is 634 nm in chloroform, while the high-energy absorption edge for the nanocrystals is 657 nm. Comparison with the absorption profiles for **1b** and **2b** confirms that no overlap occurs between the emission from the quantum dot and the absorption by the oligothiophene (Figure 5). As such, any luminescence quenching observed would not be a result of Förster energy transfer.

**Figure 6:**
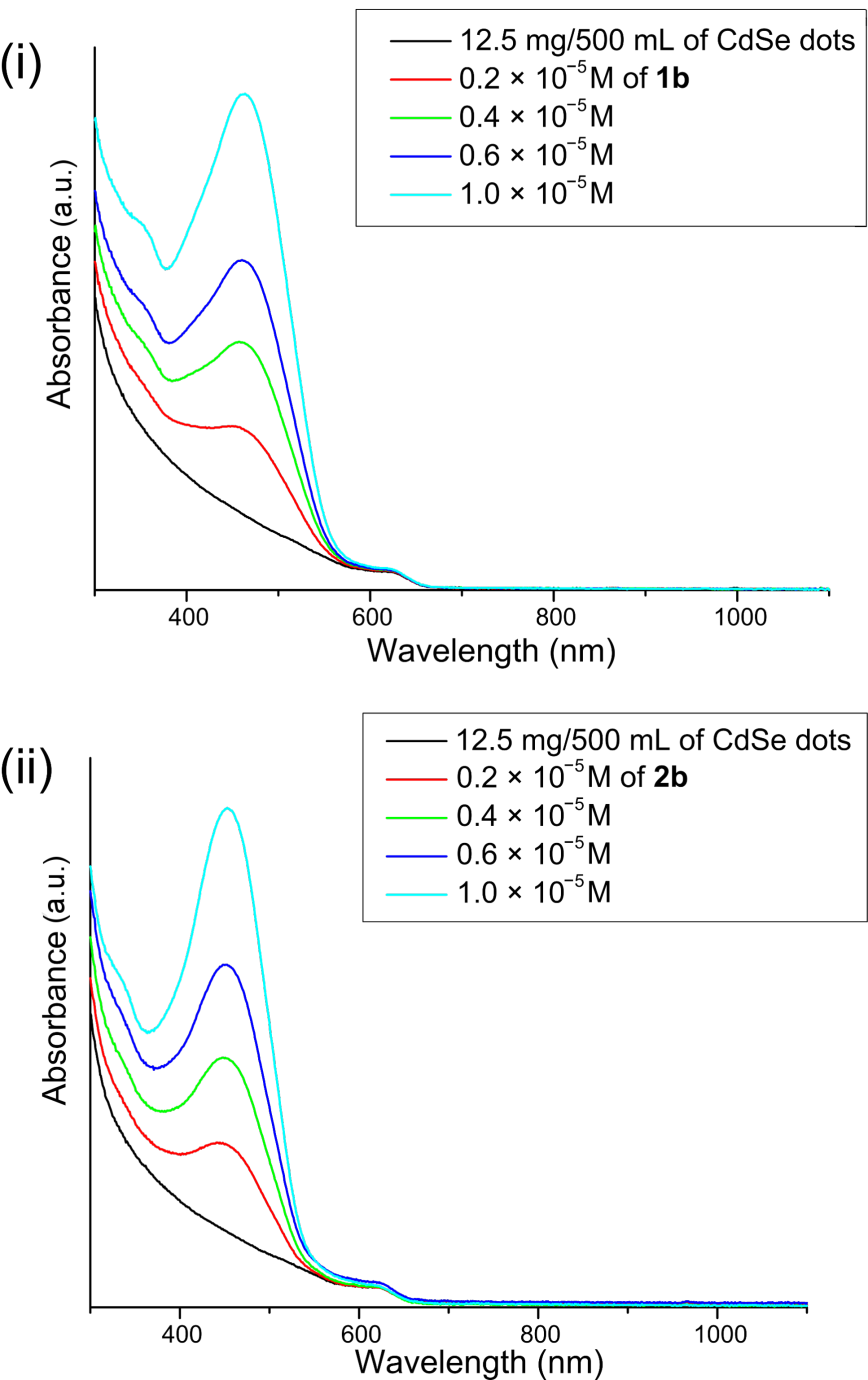
The absorption spectra of increasing sexithiophene concentration with HDA capped CdSe(ZnS) quantum dots in chloroform; (i) **1b**; (ii) **2b**.

**Figure 7:**
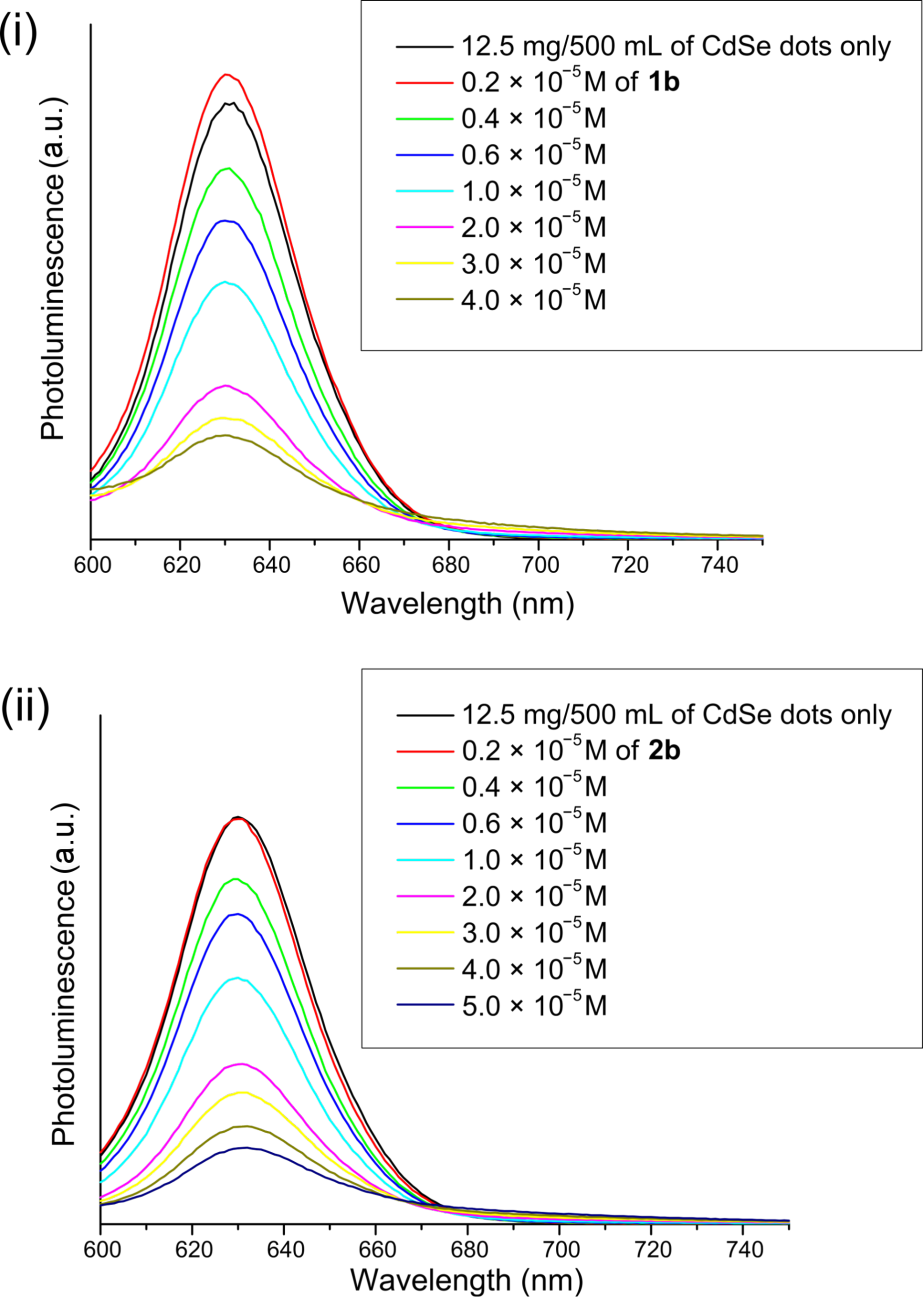
Photoluminescence quenching experiments; the effect of increasing sexithiophene concentration with HDA capped CdSe(ZnS) quantum dots, in chloroform. Excitation wavelength = 590 nm; (i) **1b**; (ii) **2b**.

The effect of each of the corresponding sexithiophenes with or without a Lewis base group (**1** or **2**) was then studied by preparing separate stock solutions of quantum dots and sexithiophenes **1b** or **2b**, of known concentration. A fixed amount of quantum dot solution was used combined with various amounts of sexithiophene **1b** or **2b** (additional pure solvent was added to maintain a fixed concentration of quantum dots). Each solution was allowed to equilibrate for 2 h, to ensure that any surface-exchange processes had reached a steady state, before the UV–vis absorption spectra were recorded (Figure 6). Although an increase in absorption was observed with increasing concentration of sexithiophene, no evidence for any ground-state interactions between the sexithiophene, **1b** or **2b**, and the CdSe(ZnS) quantum dots was observed. Next, in order to examine any possible transfer processes within the mixed solutions, photoluminescence quenching experiments were performed (Figure 7). The quantum dots were excited at 590 nm (outside the absorption region for oligothiophenes **1b** and **2b**) and the photoluminescence spectra were recorded at each concentration of **1b** and **2b**. The general trend observed was a decrease in the photoluminescence intensity with increasing sexithiophene concentration, irrespective of the presence (**1b**) or absence (**2b**) of a Lewis base (the anomaly at low concentrations of sexithiophenes is explained below). Since energy transfer can be excluded, it may be that hole transfer from the quantum dot (the HOMO of CdSe(ZnS) was reported as 6.5 eV [27]) to sexithiophenes occurs, leading to the extensive quenching observed here. A Stern–Volmer plot indicated that sexithiophenes **1b** and **2b** are equally effective (within experimental error) at quenching quantum dots (a plot of *I*_0_/*I* versus [Q] showed a slope of 1.120 ± 0.174 for **1b**, and 1.101 ± 0.114 for **2b**). As such, it is likely that both sexithiophenes **1b** and **2b**, regardless of the presence of a conventional Lewis base group, are effective in displacing HDA and quenching the luminescence of the quantum dots.

As a further comparison, terthiophene **5** was also examined for photoluminescence quenching under identical conditions to those used for sexithiophenes **1b** and **2b**. Interestingly, the addition of terthiophene **5** has no quenching effect on the luminescence of the quantum dots, indicating that the HOMO and LUMO of terthiophene **5** are misaligned with respect to the quantum dot such that the quenching does not occur. However, a slight increase in the intensity of the quantum-dot photoluminescence was observed with increasing concentrations of terthiophene **5**, indicating an effective passivation of the nonradiative decay sites [28]. At low concentration of sexithiophene **1b** (0.2 × 10^−5^ M), a slight increase in luminescence intensity was observed, whereas the luminescence intensity at the same concentration of sexithiophene **2b** remained constant. At these concentrations, the amount of sexithiophene bound to the quantum dot surface is at its lowest and a significant amount of HDA remains attached to the surface also. Since the photoluminescence is increased at these concentrations, we postulate that the sexithiophenes preferentially bind to defects on the surface of the quantum dots that otherwise act as intergap trap states for nonradiative emission.

## Conclusion

Two novel sexithiophene families, **1a**,**b** and **2a**,**b**, were synthesised and investigated as luminescence quenchers of quantum dots. Their properties were investigated by cyclic voltammetry, UV–vis absorption and spectroelectrochemistry. The addition of the terminal 4-(dimethylamino)phenyl unit in **1a** and **1b** has a noticeable effect on the optical and electronic properties of the sexithiophene, compared to the nonfunctionalised systems **2a** and **2b**, notably lowering the band gap and red-shifting the absorbance due to the increased degree of conjugation that the functionalisation affords.

Composite solutions of CdSe(ZnS) quantum dots with various concentrations of **1b** and **2b** were prepared, and the excited state interactions between the components were studied by photoluminescence spectroscopy. Upon excitation of solely the quantum dot at 590 nm (no overlap of the sexithiophene absorption and quantum dot luminescence occurs at this wavelength), both sexithiophenes **1b** and **2b** proved to be effective luminescence quenchers across a range of concentrations. It is likely that a hole-transfer mechanism operates in this process. Thus, these results indicate that sexithiophenes based on the skeleton of compound **2** are effective luminescence quenchers even without functionalisation with a Lewis base group.

## Experimental

### General

Melting points were taken on a Stuart Scientific SMP1 Melting Point apparatus and are uncorrected. ^1^H and ^13^C NMR spectra were recorded on a Varian Unity Innova instrument at 300 and 75 MHz, respectively; chemical shifts are given in ppm. Peak multiplicities are denoted by s (singlet), d (doublet), t (triplet), q (quartet) and m (multiplet) or by a combination of these dd (doublet of doublets), dt (doublet of triplets) and td (triplet of doublets), with coupling constants (*J*) given in Hz. IR spectra were recorded on an ATI Mattson Genesis Series FTIR spectrometer. Electron-impact (EI) and chemical-ionisation (CI) mass spectra were recorded on a Micromass Trio 2000 spectrometer; high-resolution mass spectra were recorded on a Kratos Concept spectrometer. Elemental analyses were obtained on a Carlo Erba Instruments EA 1108 elemental analyser. Absorption spectra were measured on a Unicam UV 300 spectrophotometer.

All CV and spectroelectrochemical measurements were performed on a CH Instruments 660 A Electrochemical Workstation with *iR* compensation, with anhydrous CH_2_Cl_2_ or acetonitrile as the solvent, aqueous Ag/AgCl as the reference electrode and platinum wire and gold disk (or ITO glass for SEC) as the counter and working electrodes, respectively. All solutions were degassed (Ar) and, where relevant, were prepared so as to contain the substrate in concentrations of ca. 10^−3^ M, together with *n*-Bu_4_NPF_6_ (0.1 M) as the supporting electrolyte. Under these conditions, the redox potential for the FcH/FcH^+^ couple was +0.48 V (CH_2_Cl_2_, versus Ag/AgCl).

The photoluminescence properties of the oligothiophene and CdSe(ZnS) quantum dot blends were measured on a Spex FluoroMax instrument with a xenon lamp (150 W) and a 152 P photomultiplier tube as a detector. Spectra were obtained with the slits set at 5 nm and with an integration time of 1 s. The samples were placed in quartz cuvettes (1 cm path length). The wavelength of excitation is indicated in the text.

The compounds **2a**, **2b** and **3** were prepared according to our previously reported procedure [14].

#### 5-(5-Bromothiophen-2-yl)-7-(5-hexylthiophen-2-yl)-2,3-dihydrothieno[3,4-*b*][1,4]dithiine (**4a**)

To a solution of **3a** (0.62 g, 1.47 mmol) in tetrahydrofuran/glacial acetic acid (1:1 v/v, 40 mL), was added *N*-bromosuccinimide (0.26 g, 1.47 mmol) portionwise, in the dark under rapid stirring. The reaction mixture was stirred for 2 h after which water (40 mL) was added. The product was then extracted into dichloromethane (2 × 30 mL), washed with saturated sodium hydrogencarbonate (2 × 30 mL) and dried (MgSO_4_). The solvent was removed under reduced pressure to afford **4a** as a dark green oil (0.71 g, 97%); ^1^H NMR (CDCl_3_) δ 7.14 (d, *J* = 3.7, 1H), 7.05 (s, 2H), 6.78 (d, *J* = 3.7, 1H), 3.30 (s, 4H), 2.85 (t, *J* = 6.6, 2H), 1.73 (q, 2H), 1.36 (m, 6H), 0.93 (t, *J* = 6.7, 3H); ^13^C NMR (CDCl_3_) δ 147.4, 136.7, 132.0, 130.4, 128.7, 126.5, 126.2, 124.8, 124.3, 123.1, 112.9, 31.8, 30.4, 29.1, 28.6, 28.5, 22.8, 14.4; MS (TOF, EI^+^) *m*/*z*: 519 (M + NH_4_^+^, 31%), 517 (M + NH_4_^+^, 28%), 503 (M + H^+^, 100%), 501 (M + H^+^, 95%); FTIR (10 scan) ν/cm^−1^: 2952, 2924, 2853, 1490, 1462, 1411, 1275, 1036; HRMS (TOF, EI^+^): calcd for C_20_H_21_BrS_5_ + H^+^, 500.9509; found, 500.9676.

#### 5-(5-Bromothiophen-2-yl)-7-(5-methylthiophen-2-yl)-2,3-dihydrothieno[3,4-*b*][1,4]dithiine (**4b**)

Compound **4b** was prepared as described for **4a** from compound **3b** (0.89 g, 2.50 mmol), in tetrahydrofuran/glacial acetic acid (1:1 v/v, 40 mL), and *N*-bromosuccinimide (0.45 g, 2.50 mmol) to afford **4b** as a yellow solid (0.96 g, 88%); mp 93–96 °C; ^1^H NMR (CDCl_3_) δ 7.14 (d, *J* = 3.7, 1H), 7.06 (s, 2H), 6.78 (dd, *J* = 1.0 and 3.7, 1H), 3.31 (s, 4H), 2.56 (s, 3H); ^13^C NMR (CDCl_3_) δ 141.2, 136.6, 132.3, 130.4, 129.4, 128.5, 126.8, 126.2, 126.1, 124.3, 123.2, 112.9, 28.6, 28.5, 15.6; MS (EI) *m*/*z*: 432 (M^+^, 16%), 430 (M^+^, 13%), 352 (11%); MS (CI) *m*/*z*: 433 (M + H^+^, 41%), 431 (M + H^+^, 37%), 353 (100%); FTIR (KBr) ν/cm^−1^: 2945, 2908, 1655, 1489, 1412, 1276, 1217, 1056; Anal. calcd for C_14_H_8_O_2_S_5_: C, 41.76; H, 2.57; S, 37.16; Br, 18.52%; found: C, 41.62; H, 2.27; S, 37.20; Br, 18.30%.

#### 4,5-Bis-(hydroxythiophene-2-yl-methyl)-[1,3]dithiole-2-thione (**A**) [29]

A solution of 1,3-dithiole-2-thione (4.00 g, 29.8 mmol) in dry tetrahydrofuran (250 mL) was cooled to −78 °C under dry nitrogen. Lithium diisopropylamide mono(tetrahydrofuran) (1.5 M in cyclohexanes, 21.9 mL, 32.9 mmol) was added and the mixture was stirred for 20 minutes after which time thiophene-2-carboxaldehyde (3.1 mL, 32.9 mmol) was added and the mixture stirred for a further 10 min. Another portion of lithium diisopropylamide mono(tetrahydrofuran) (1.5 M in cyclohexanes, 21.9 mL, 32.9 mmol) was added and the mixture was stirred for 15 min after which time thiophene-2-carboxaldehyde (3.1 mL, 32.9 mmol) was added and the mixture was stirred for a further 5 min. The reaction mixture was stirred for a further 1.5 h whilst being allowed to warm to room temperature. Saturated sodium hydrogencarbonate (150 mL) was added and the organic phase was removed. The aqueous phase was washed with dichloromethane (3 × 50 mL) and the combined organic extracts were dried (MgSO_4_). The solvent was removed under reduced pressure and the product was isolated by column chromatography (silica, petroleum ether (40–60 °C)/dichloromethane (1:1 v/v) to remove the starting materials and impurities, then ethyl acetate to remove the product) to afford **A** as a red oil (~10.1 g; product unstable, no data).

#### [5-(Thiophene-2-carbonyl)-2-thioxo-[1,3]dithiol-4-yl]thiophen-2-yl-methanone (**B**) [29]

To a solution of the diol **A** (10.1 g), in dichloromethane (250 mL), manganese dioxide (10× excess w/w, ~101 g) was added portionwise and the mixture was stirred for approximately 2 min. The mixture was filtered through a silica plug (eluted with dichloromethane) and the solvent removed under reduced pressure to afford **B** as a yellow solid (9.50 g, 90% from 1,3-dithiole-2-thione **3**); mp 91–92 °C (lit. [29] mp 92 °C); ^1^H NMR (CDCl_3_) δ 7.76 (dd, *J* = 1.1 and 4.9, 2H), 7.72 (dd, *J* = 1.1 and 4.0, 2H), 7.14 (t, *J* = 4.4, 2H); MS (EI) *m*/*z*: 354 (M^+^, 75%), 278 (12%), 250 (13%); MS (CI) *m*/*z*: 372 (M + NH_4_^+^, 100%), 355 (M + H^+^, 58%), 281 (21%).

#### 4,5-Bis(thiophene-2-carbonyl)[1,3]dithiole-2-one (**C**) [30]

To a solution of the diketone **B** (9.5 g, 26.8 mmol) in dichloromethane/glacial acetic acid (3:1 v/v, 200 mL), was added mercuric acetate (11.98 g, 37.6 mmol). The mixture was stirred at room temperature for 16 h and filtered through a silica plug (eluting with dichloromethane). The organic extract was washed with water (2 × 100 mL) and saturated sodium hydrogencarbonate (2 × 100 mL), and was dried (MgSO_4_). The solvent was removed under reduced pressure to afford **C** as an off-white solid (7.35 g, 81%); mp 130–131 °C (lit. [30] mp 130–132 °C); ^1^H NMR (CDCl_3_) δ 7.75 (dd, *J* = 1.1 and 4.9, 2H), 7.73 (dd, *J* = 1.1 and 4.0, 2H), 7.14 (t, *J* = 4.4, 2H); MS (EI) *m*/*z*: 338 (M^+^, 19%); MS (CI) *m*/*z*: 356 (M + NH_4_^+^, 100%), 339 (M + H^+^, 19%).

#### 4,6-Di-thiophen-2-yl-thieno[3,4-*d*][1,3]dithiol-2-one (**D**) [30]

A mixture of the tricarbonyl **C** (3.68 g, 10.9 mmol), sodium hydrogencarbonate (4.57 g, 54.5 mmol) and phosphorus pentasulfide (24.2 g, 54.5 mmol) in 1,4-dioxane (150 mL) was stirred whilst the temperature was raised to 90 °C. This temperature was maintained for 3 h. The mixture was cooled, water (200 mL) was added portionwise (CAUTION! H_2_S and CO_2_ evolution) and the suspension was stirred overnight. After cooling, the mixture was filtered, washed with boiling water and dried in vacuo to afford the crude product*.* The crude product was dissolved in minimal hot chloroform, dried (MgSO_4_), stirred with decolourising charcoal for 30 min and then filtered through a silica plug (eluting with chloroform). The solvent was reduced in volume and the product isolated by precipitation with petroleum ether (40–60 °C), to afford **D** as an orange/yellow solid (2.17 g, 59%); mp 169–170 °C (lit. [30] mp 170–172 °C); ^1^H NMR (CDCl_3_) δ 7.42 (dd, *J* = 1.1 and 5.1, 2H), 7.28 (dd, *J* = 1.1 and 3.7, 2H), 7.15 (t, *J* = 4.4, 2H); MS (EI) *m*/*z*: 338 (M^+^, 51%), 310 (19%), 265 (19%); MS (CI) *m*/*z*: 356 (M + NH_4_^+^, 5%), 339 (M + H^+^, 100%).

#### 5,7-Dithiophen-2-yl-2,3-dihydrothieno[3,4-*b*][1,4]dithiine (**5**) [30]

To a solution of the terthiophene **D** (2.17 g, 6.4 mmol) in dry tetrahydrofuran (150 mL), was added sodium ethoxide (0.2 M solution in ethanol, 70.6 mL, 14.1 mmol) under dry nitrogen. The reaction mixture was stirred for 30 min after which time 1,2-dibromoethane (0.55 mL, 6.4 mmol) was added and the mixture was stirred at room temperature for a further 16 h. The reaction mixture was filtered through a silica plug (eluting with tetrahydrofuran) and the solvent removed under reduced pressure to leave the crude product. The crude product was dissolved in chloroform, stirred with decolourising charcoal for 30 min and then filtered through a silica plug (eluting with chloroform). The solvents were removed under reduced pressure to afford **5** as a yellow solid (1.94 g, 66%); mp 140–141 °C (lit. [30] mp 140–142 °C); ^1^H NMR (CDCl_3_) δ 7.39 (dd, *J* = 1.2 and 5.1, 2H), 7.36 (dd, *J* = 1.2 and 3.7, 2H), 7.13 (t, *J* = 4.5, 2H), 3.34 (s, 4H); MS (EI) *m*/*z*: 338 (M^+^, 18%); MS (CI) *m*/*z*: 339 (M + H^+^, 100%).

#### 5,7-Bis-(5-bromothiophen-2-yl)-2,3-dihydrothieno[3,4-*b*][1,4]dithiine (**6**)

To a solution of the terthiophene **5** (1.94 g, 5.2 mmol), in tetrahydrofuran/glacial acetic acid (1:1 v/v, 100 mL) was added *N*-bromosuccinimide (2.04 g, 11.5 mmol) portionwise, in the dark and under rapid stirring. The reaction mixture was stirred for 2 h after which water (150 mL) was added and the resulting precipitate was collected by filtration. The crude product was redissolved in chloroform and dried (MgSO_4_). The chloroform was reduced in volume and the product was isolated by precipitation with petroleum ether (40–60 °C) to afford **6** as a yellow solid (2.57 g, 91%); mp 156–157 °C; ^1^H NMR (CDCl_3_) δ 7.06 (s, 4H), 3.33 (s, 4H); MS (APCI^+^) *m*/*z*: 496 (MH^+^, 21%), 418 (100%); MS (APCI^−^) *m*/*z*: 468 ([M – C_2_H_4_]^−^, 100%); FTIR (KBr) ν/cm^−1^: 2926, 1655, 1560, 1508, 1476, 1419, 1272, 1217; Anal. calcd for C_14_H_8_Br_2_S_5_: C, 33.88; H, 1.62; S, 32.30; Br, 32.20%; found: C, 34.14; H, 1.42; S, 32.64; Br, 31.98%.

#### 5-Bromo-5'''''-hexyl-3',3'''',4',4''''-ethylenedithio-2,2';5',2'';5'',2''';5''',2'''';5'''',2'''''-sexithiophene (**7a**)

To a solution of compound **4a** (0.71 g, 1.42 mmol), in dry tetrahydrofuran (10 mL), under dry nitrogen and at −78 °C, was added *n*-butyllithium (2.5 M in hexanes, 0.74 mL, 1.85 mmol) and the mixture was stirred for 45 min. Then, to this a solution of zinc(II)chloride (0.25 g, 1.85 mmol) in dry tetrahydrofuran (20 mL) prepared under dry nitrogen was added by cannula, before being allowed to warm to room temperature. This was then added to a solution of compound **6** (2.10 g, 4.26 mmol) and tetrakis(triphenylphosphine)palladium(0) (0.12 g, 0.099 mmol) in dry tetrahydrofuran (50 mL) under dry nitrogen, and heated under reflux for 16 h. The solution was allowed to cool and the crude product precipitated with petroleum ether (40–60 °C). The crude product was redissolved in a minimal amount of chloroform, reduced in volume and reprecipitated with petroleum ether (40–60 °C). The crude product was precipitated a number of times from chloroform to remove the majority of the excess of compound **6** used. The traces of **6** were removed by washing the solid (by using soxhlet extraction apparatus) with methanol. The crude product was then collected with chloroform and isolated by precipitation. The product was isolated by column chromatography (silica, toluene) and then precipitation to afford **7a** as a red solid (0.47 g, 40%); mp 177 °C (by DSC); ^1^H NMR (CDCl_3_) δ 7.27 (m, 2H), 7.20 (dd, *J* = 0.8 and 3.9, 2H), 7.17 (d, *J* = 3.5, 1H), 7.08 (m, 2H), 6.80 (d, *J* = 3.7, 1H), 3.36 (s, 8H), 2.86 (t, *J* = 7.7, 2H), 1.74 (q, 2H), 1.37 (m, 6H), 0.94 (t, *J =* 6*.*9, 3H); MS (MALDI-TOF) *m*/*z*: 838 (M^+^, 100%); FTIR (KBr) ν/cm^−1^: 2924, 2855, 1634, 1266, 1161, 1040; Anal. calcd for C_34_H_29_BrS_10_: C, 48.72; H, 3.49; Br, 9.53%; found: C, 48.31; H, 3.22; Br, 9.05%.

#### 5-Bromo-5'''''-methyl-3',3'''',4',4''''-ethylenedithio-2,2';5',2'';5'',2''';5''',2'''';5'''',2'''''-sexithiophene (**7b**)

Compound **7b** was prepared as described for **7a** from compound **4b** (0.66 g, 1.5 mmol) in dry tetrahydrofuran (20 mL), *n*-butyllithium (2.5 M in hexanes, 0.80 mL, 2.0 mmol), zinc(II)chloride (0.27 g, 2.0 mmol) in dry tetrahydrofuran (20 mL), compound **6** (2.27 g, 4.60 mmol) and tetrakis(triphenylphosphine)palladium(0) (0.13 g, 0.11 mmol) in dry tetrahydrofuran (60 mL). The product was isolated by column chromatography (silica, toluene) and then precipitation to afford **7b** as a red solid (0.23 g, 20%); mp 195 °C (by DSC); ^1^H NMR (CDCl_3_) δ 7.26 (m, 2H), 7.20 (d, *J* = 4.0, 2H), 7.15 (d, *J* = 3.5, 1H), 7.08 (m, 2H), 6.80 (d, *J* = 3.5, 1H), 3.36 (s, 8H), 2.55 (s, 3H); MS (MALDI-TOF) *m*/*z*: 769 (M^+^, 68%), 767 (M^+^, 100%); FTIR (KBr) ν/cm^−1^: 2910, 1619, 1476, 1407, 1272, 1213, 1159, 1056; Anal. calcd for C_29_H_19_BrS_10_: C, 45.35; H, 2.49; Br, 10.40%; found: C, 45.08; H, 2.13; Br, 10.73%.

#### 5-(4-[Dimethylamino]phenyl)-5'''''-hexyl-3',3'''',4',4''''-ethylenedithio-2,2';5',2'';5'',2''';5''',2'''';5'''',2'''''-sexithiophene (**1a**)

To a solution of **7a** (136 mg, 0.16 mmol) and tetrakis(triphenylphosphine)palladium(0) (10 mg, 0.008 mmol) in toluene (20 mL) under dry nitrogen, was added, subsequently, a suspension of 4-(dimethylamino)phenylboronic acid (35 mg, 0.21 mmol) in ethanol (25 mL) and a solution of anhydrous sodium carbonate (45 mg, 0.42 mmol) in water (5 mL). The mixture was then heated under reflux for 16 h. Then, the solution was allowed to cool to room temperature and toluene (30 mL) was added to dilute the organic phase. The aqueous phase was removed and extracted with toluene (3 × 30 mL). The organic phases were combined and dried (MgSO_4_). The solvents were reduced in volume and the crude product was precipitated with petroleum ether (40–60 °C). The product was isolated by column chromatography (neutral alumina, toluene/ethyl acetate 9:1 (v/v) with gradual change to ethyl acetate). The solvents were reduced in volume and the product was precipitated with petroleum ether (40–60 °C) to afford **1a** as a red solid (40 mg, 28%); mp 192–194 °C; ^1^H NMR (CDCl_3_) δ 7.55 (d, *J* = 8.6, 2H), 7.28 (m, 2H), 7.22 (m, 3H), 7.17 (d, *J* = 4.0, 2H), 6.78 (m, 3H), 3.36 (s, 8H), 3.04 (s, 6H), 2.86 (t, *J* = 7.5, 2H), 1.74 (q, 2H), 1.37 (m, 6H), 0.92 (t, *J =* 6*.*9, 3H); MS (APCI^+^) *m*/*z*: 895 (M + NH_4_^+^, 100%), 878 (M + H^+^, 53%); MS (APCI^−^) *m*/*z*: 848 ([M – 2CH_3_]^−^, 100%); FTIR (KBr) ν/cm^−1^: 3055, 2916, 2850, 1604, 1419, 1342, 1265, 1172, 1049; HRMS (TOF, EI^+^): calcd for C_42_H_39_NS_10_ + H^+^, 878.0362; found, 878.0372.

#### 5-(4-[dimethylamino]phenyl)-5'''''-methyl-3',3'''',4',4''''-ethylenedithio-2,2';5',2'';5'',2''';5''',2'''';5'''',2'''''-sexithiophene (**1b**)

Compound **1b** was prepared as described for **1a** from compound **7b** (78 mg, 0.10 mmol) in toluene (20 mL), tetrakis(triphenylphosphine)palladium(0) (6 mg, 0.005 mmol), 4-(dimethylamino)phenylboronic acid) (22 mg, 0.13 mmol) in ethanol (25 mL) and anhydrous sodium carbonate (28 mg, 0.26 mmol) in water (5 mL). The product was isolated by column chromatography (neutral alumina, toluene/ethyl acetate 9:1 (v/v) with a gradual change to ethyl acetate). The solvents were reduced in volume and the product precipitated with petroleum ether (40–60 °C) to afford **1b** as a red solid (36 mg, 44%); mp 152–155 °C; ^1^H NMR (CDCl_3_) δ 7.55 (d, *J* = 8.78, 2H), 7.26 (m, 2H), 7.22 (m, 3H), 7.17 (d, *J* = 4.0, 1H), 7.15 (d, *J* = 4.0, 1H), 6.77 (m, 3H), 3.36 (s, 8H), 3.04 (s, 6H), 2.56 (s, 3H); MS (APCI^+^) *m*/*z*: 808 (MH^+^, 100%); FTIR (KBr) ν/cm^−1^: 3055, 2916, 1605, 1419, 1342, 1265, 1157, 1049; HRMS (TOF, EI^+^): calcd for C_37_H_29_NS_10_ + H^+^, 806.9502; found, 806.9494.
